# Health Care Professionals’ Experience of a Digital Tool for Patient Exchange, Anamnesis, and Triage in Primary Care: Qualitative Study

**DOI:** 10.2196/21698

**Published:** 2020-12-14

**Authors:** Ann Catrine Eldh, Annette Sverker, Preben Bendtsen, Evalill Nilsson

**Affiliations:** 1 Department of Health, Medicine and Caring Sciences Linköping University Linköping Sweden; 2 Department of Public Health and Caring Sciences Uppsala University Uppsala Sweden; 3 Department of Rehabilitation Medicine and Department of Health, Medicine and Caring Sciences Linköping University Linköping Sweden; 4 Department of Medical Specialists in Motala and Department of Health, Medicine and Caring Sciences Linköping University Linköping Sweden; 5 e-Health Institute Department of Medicine and Optometry Linneaus University Kalmar Sweden

**Keywords:** communication, content analysis, eHealth, telemedicine, digital technology, interviews, primary health care, qualitative research

## Abstract

**Background:**

Despite a growing body of knowledge about eHealth innovations, there is still limited understanding of the implementation of such tools in everyday primary care.

**Objective:**

The objective of our study was to describe health care staff’s experience with a digital communication system intended for patient-staff encounters via a digital route in primary care.

**Methods:**

In this qualitative study we conducted 21 individual interviews with staff at 5 primary care centers in Sweden that had used a digital communication system for 6 months. The interviews were guided by narrative queries, transcribed verbatim, and subjected to content analysis.

**Results:**

While the digital communication system was easy to grasp, it was nevertheless complex to use, affecting both staffing and routines for communicating with patients, and documenting contacts. Templates strengthened equivalent procedures for patients but dictated a certain level of health and digital literacy for accuracy. Although patients expected a chat to be synchronous, asynchronous communication was extended over time. The system for digital communication benefited assessments and enabled more efficient use of resources, such as staff. On the other hand, telephone contact was faster and better for certain purposes, especially when the patient’s voice itself provided data. However, many primary care patients, particularly younger ones, expected digital routes for contact. To match preferences for communicating to a place and time that suited patients was significant; staff were willing to accept some nuisance from a suboptimal service—at least for a while—if it procured patient satisfaction. A team effort, including engaged managers, scaffolded the implementation process, whereas being subjected to a trial without likely success erected barriers.

**Conclusions:**

A digital communication system introduced in regular primary care involved complexity beyond merely learning how to manage the tool. Rather, it affected routines and required that both the team and the context were addressed. Further knowledge is needed about what factors facilitate implementation, and how. This study suggested including ethical perspectives on eHealth tools, providing an important but novel aspect of implementation.

## Introduction

### Background

In many countries, primary care providers struggle to meet the needs and demands of increasing numbers of patients seeking their services [1]. In this case, primary care implies the entry point or opening contact with health care, provided by outpatient services located within the community but organized by the regional or a private health care organization. Lack of access to such care can have a negative impact on both individuals and populations; thus, there is a need for more effective routes to supply frontline health services to large and diverse populations [2].

During the last decade, several innovations for online communication with health care providers have emerged, such as video links, text messaging, email, and web-based portals. Despite progress, evidence for the benefits of introducing digital patient-professional communication within primary care is incomplete [3]. For example, although digital communication seems to enhance patient satisfaction, data about the impact on health outcomes are limited, and there is no consensus about the optimal design of digital communication services [3]. A systematic review of e-consultations in primary care identified 5 themes: patient access, patient outcomes, workforce issues, governance and safety, and willingness to adopt and sustain the service [4]. Nevertheless, the implementation process of eHealth innovations has attracted limited attention, despite its importance for progress [5]. Further attention should thus include end-user perspectives: a progression toward efficient services includes advancing technological innovations that facilitate patients, health care professionals, and organizations [6,7].

Within Swedish health care, the initial provision of digital primary care services by for-profit companies commenced a few years ago [8], although lately access to digital services through regular primary care providers has been increasing. Swedish health care is heavily decentralized, and thus mainly organized independently by the 21 regions of the nation. Currently, several regions are trialing digital services in primary care; one of these is Flow, a Swedish commercial digital communication service [9]. In 2019, a typical region tested the system, advertising it to patients through the general health care website or standard telephone service of their regular primary care provider as an alternative to applying for a consultation with the triage nurse via telephone. When choosing the pilot digital communication route, the patients received access via a link, identifying themselves via secure electronic personal identification.

Once patients had registered with the system, they were asked structured questions about the reason for the contact (equivalent to matter or issue), composing an anamnesis by means of extensive medically approved templates consisting of drop-down menu options tied to fit captions and free-text alternatives. While the patients could post their issues at any time, they were informed that the service was staffed only during the daytime (8:00-15:00), Monday through Friday. Submitted issues were posted as a case with the patients’ primary care center, where a triage nurse responded within 2 hours, during office hours, initiating an asynchronous chat. The nurse could thus post further questions to patients (which they could respond to whenever appropriate) or refer the case to another staff member on the team (such as a secretary, for administrative issues, or a physician). Patients had the final say about when to close the chat, and the conversation was then manually inserted by the health care professional into patients’ standard electronic health records.

### Objective

This innovation, like equivalent digital tools, is promoted as an efficient use of resources in the often-burdened field of primary care. Yet the possibility of combining an online medical form with subsequent text messaging communication has been found to be suboptimally used [2]. Primary care, like all health care services, should optimize quality and efficiency, offering user-friendly options. Further understanding about what constitutes the ideal design, implementation, and use of digital services is needed [10,11]. In this study, we took an end-user perspective, aimed at conveying health care staff’s experiences with a digital communication system used for patient-staff encounters via asynchronous chat in Swedish primary care.

## Methods

### Design and Setting

The study design was explorative and descriptive. We interviewed health care staff about halfway into the 1-year pilot period following a regional decision to test the digital communication system. We were independent of the project management but were assigned to investigate the implementation. A total of 5 primary care centers in a typical region of southeast Sweden had agreed to test the digital service, which was free of charge during the pilot period. The primary care centers were representative of Sweden and the region, including urban as well as larger and smaller community settings. The staff who were meant to use the system in their everyday practice and the primary care center managers from the start had received a concise training session (less than half a day) led by a project manager; staff who joined later were introduced to the system by their colleagues in each primary care center. The primary care centers then separately organized their use of the digital communication service; for instance, 1 of the 5 primary care centers included physicians in addition to the nurses and administrators who constituted the backbone of the service.

### Sample

The project manager provided us with contact information for the health care staff who were using the digital communication system, collated by the primary care center managers. We identified a sample of 4 potential study participants from each primary center: (1) a manager, (2) at least one district nurse or registered nurse, or (3) a district or registered nurse and at least one physician, and (4) at least one secretary. We restricted the inclusion of managers, physicians, or district nurses—that is, nurse specialists—as not all primary care centers had these groups of professionals engaged in the test. We contacted the potential respondents via email with information about the study and a request to let us know whether they were willing to participate. We sent 1 reminder where necessary. If we received no response or a refusal, we identified a matching substitute in the primary care center and repeated the informed-consent process until we achieved the preferred sample. If several staff were available, we made a strategic selection to match the above roles, but also to consider a fair representation of gender in relation to roles across all respondents.

### Procedure

#### Data Collection

All interviews were individual and conducted by telephone; a researcher called at an agreed-upon date and time, recapped the information about the study, and asked for consent. All participants provided verbal informed consent.

The interviews were performed by either of 2 researchers on the team: the first researcher (ACE) has extensive experience in qualitative methods and mentored a second researcher (EN), who is a well-rehearsed scholar. Besides data collection sessions, consistency was enhanced by an agreed-upon semistructured interview guide compiled for the study. This comprised 4 main areas related to the digital communication service: the primary care process, the implementation process, the digital communication system itself, and the patient-professional relationship. The guide had been developed for and tested in a previous study addressing patients’ experience; it was found to facilitate a narrative regarding the core issues of primary care interactions using the digital communication system, and we thus applied it after a minor linguistic adaptation to address staff experience. We used probes only if the participant did not freely elaborate on particulars, such as training, in his or her narrative. We asked 3 final demographic questions: age, gender, and how confident the respondent was about managing everyday issues with their computer or smartphone.

All interview recordings were transcribed verbatim by a skilled secretarial service (Literatim, Stockholm, Sweden) prior to the analysis. A total of 21 individuals participated in the study: 19 women, and 2 men, age range 25 to 67 years (median 44 years). Most (n=16) were “always or almost always” able to manage their computer and smartphone on their own (with 5 “usually” managing and none opting for the “seldom” or “never/almost never” alternatives). The interviews lasted between 14 and 41 minutes, mean 22 minutes. The transcripts constituted 126 pages of single-spaced text. We considered data saturation [12], and the interviewers maintained a dialogue during the data collection period, identifying that we obtained a rich set of illustrations across all interview queries from the interviews. We did not report any data back to the participants or the primary care centers.

#### Data Analysis

The analysis followed the trajectory of content analysis, as described by Elo and Kyngäs [13].

First, all 4 researchers separately read all transcribed interviews, forming an individual naïve understanding, then assembled and discussed them in the team.

Second, the subsequent structured analysis was informed by the identification of 8 main components of the mutual naïve understanding. We used these components to organize the analysis, meaning that we identified units from all the interviews and assembled them according to these codes, and we separated units encompassing meaning beyond 1 component or code into 2 (or more, as appropriate) [14]. Each interview text was thus once again read and reread separately, and all meaning units were assembled for each component. We further analyzed these texts by using an inductive approach to form categories and subcategories, thus procuring a shared human experience [15].

Third, we abstracted the categories and corresponding subcategories into a text that reflected a thorough understanding of the experience of using a digital communication system while working as a health care member of staff in primary care, also known as a comprehensive understanding [13].

We selected quotes to illuminate the findings and make it easier for others to grasp the main content, in terms of everyday language and experience. The quotes were translated into English by the researchers, preserving a unique meaning, and checked for grammatical accuracy by a proofreading service (Sprakservice, Malmö, Sweden).

Throughout both the structured analysis and the comprehensive understanding phases, we repeatedly discussed the evolving findings as a team [16].

## Results

### Comprehensive Understanding

The comprehensive understanding of the experience of the digital communication system in primary care incorporated 4 global themes: the innovation itself, the implementation process (addressing the device and context changes), the barriers (incorporating patient and staff needs and skills), and indicating a work in progress, where both the outcomes and, in particular, the benefits vary. The comprehensive understanding was scaffolded by the following structured analysis, with the categories marked in italics, and concluding with a summary. Refer to Multimedia Appendix 1 for an overview of the global elements, categories, and subcategories.

#### The Innovation

The digital communication system *required particular patient skills*, entailing working on an internet-connected computer, iPad, or smartphone and requiring a basic awareness of terms related to health and health care. In order to complete the templates, not skipping any mandatory details, the patient needed to follow the path designated for each trajectory, describing only symptoms that were related to the reason for contact, and specifying while not exaggerating. This also called for skills in expressing oneself in writing. Likewise, the ability of health care staff to phrase articulate and prompt responses and further queries in the chat dialogue affected the liability but also required more time than a phone conversation.

Many patients who seek advice for upper tract infections tick the box for having chest pain. For me as a professional, it [chest pain] indicates something severe. But then it turns out to be, like “it hurts a little when I cough.”

Interviewee no 7

Besides skills, the digital communication system also required *patient access to devices* (an internet-connected computer, iPad, or smartphone), along with access to and the skills to use an e-ID. Thus, staff considered that the digital communication system was mainly beneficial to the young and to resourceful groups of patients, gaining a further route for contact and thus quicker communication. Empowering those in society who can navigate the system, the digital communication system may *buttress patient inequity*, professionals suggested.

The digital communication system offered what was advertised as the potential to chat with the primary care center, which some patients interpreted as a concurrent service, although it was actually asynchronous. The disparate limits on further dialogue, whereby staff are expected to respond within a couple of hours during daytime, while the patient is able to respond at any time for several days, caused lags in the *asynchronous chat*.

#### Implementation: The Device and the Change Process

The digital communication system was accompanied by *varying preimplementation training*; the staff claimed to have had either a common training by the company or by a particular assigned project instructor, or by a colleague who had been trained by the instructor. This was either in-house consecutive guidance or a joint event for the center’s team. The training, much like the decision to join the project, varied between the primary care centers: either the entire team was engaged in the dialogue regarding the digital service pilot, or it was a sole management decision. Moreover, the attitude of the manager influenced whether and how innovations spread through the team, whereas a mere order impeded further discussion.

We’ve piloted before and are pretty eager to trial, particularly when it comes to eHealth and stuff. But we’re used to more support.

Interviewee no 3

Although the digital communication system was simple to use, it was considered to have been *manufactured for a team*, requiring the routines it affects to be identified. This takes time, as does changing the routines to make best use of the innovation. Across the primary care centers, the nurses were in charge of triaging incoming cases; local routines were set up for how the nurses should use the chat function, allocating resources from the prevailing telephone service but with no corresponding increase in staffing. Rather, nurses took turns in managing the digital system, communicating with patients and surveying the errands, the latter including the follow-up on cases not resolved during each shift. Secretaries were invited to manage administrative issues and physicians managed the complex medical issues. While 1 of the 5 primary care centers included some physicians in *a team effort* at trialing the digital communication system, the nurses could direct the more complex medical issues straight to another professional within the system. Though physicians were portrayed as reluctant to engage, in all centers they were willing to confer about cases that the nurses brought up with them.

They [the nurses] chat with the patients and resolve the issues that they can, and if they need assistance from a doctor, they send on the thread.

Interviewee no 1

Settling the digital communication system into everyday practice meant more than getting it up and running: the implementation of the innovation drew attention to how complex patients’ issues can be and how the digital communication system supported this complexity, or not. In response to patients who found ways around the mandatory features of the innovation, staff identified ways to uphold safe procedures. Furthermore, staff remodeled their routines along the way, identifying and agreeing upon new standards. Thus, *implementation took time* beyond the actual training and launching of the digital communication system. Yet it safeguarded a structure that was invented for and scaffolded the center, *inventing keys along the way*, in terms of both altering routines and learning how to take advantage of the digital communication system. At the point when the interviews were conducted, the primary care centers were pondering whether and how the innovation worked to their satisfaction.

The routines, they need to settle. Like, we became better at telling the patients that “now, this is another [health] issue that you’re asking about, so you have to initiate a new thread.”

Interviewee no 19

#### The Barriers: Patient and Staff Needs and Skills

The *lack of synchronicity between systems* resulted in a lack of connection between them, requiring that the staff manually register data from chat to the patient record system. Further, the patients were using several routes (eg, both telephone services and the digital communication service), thus occupying further resources for the same issue. As different staff members operated different services, the realization that multiple professionals were engaged with the same patient was purely coincidental. Furthermore, the *writing took time*, as there were no templates for replies and writing takes longer than talking over the phone. Additionally, once one was writing in the chat function, the lag made registration more time consuming.

Patient *matters derived from a wide range of aspects*, and although some issues were considered better managed due to the digital communication system, it was difficult to chat with patients about mental health issues. On the one hand, some patients preferred the more anonymous digital chat, which also offered an opportunity to write at any time of day, for sharing moods and feelings. On the other hand, it was harder to decipher details in text, but a verbal dialogue was more beneficial for such issues.

The extensive text bulk produced by a chat meant *the staff lacked an overview*. Staff operated the digital communication system for a shift at a time, and the patients did not have a set time limit within which to respond, extending the communication to weeks in some cases. Regardless of whether the nurses followed up their assigned chats or dispatched their ongoing chats after each shift, prolonged chats were considered risky, requiring either multitasking or cases being assessed differently, and thus muddling the response to the patient.

We need someone online who can respond in real time. But we don’t have that kind of numbers [of incoming issues] to justify such resources.

Interviewee no 17

A primary uncertainty, however, originated from *missing data*; because of a lack of understanding of the necessity for a complete anamnesis, patients could skip mandatory items by means of alternative characters (eg, forcing the template by writing a period only). Furthermore, hearing a patient speak sometimes makes a big difference in achieving a correct assessment, covering a variety of aspects such as mood, confusion, a sore throat, or breathing problems.

It’s hard enough to triage over the phone, but at least you have the voice to guide you, the tone, and additional sounds and such. Here, it’s only the text.

Interviewee no 5

#### The Outcomes and Pros: A Work in Progress

The *prime advantages* of the digital system included the possibility for patients to upload photos, for example, on skin problems, such as rashes and eczema. Although none of the primary care centers used video chat, the triage was considered safer and more prompt with pictures, often saving patients a visit to the primary care center. This further sustained a safe environment because, for example, parents could be counselled over the chat when a photo had confirmed a child with chickenpox, rather than showing up at the center and risking contagion.

The photos, that’s great. It’s been all kinds of things: noses—are they broken or not? Is this foot swollen or not, and how to deal with it.

Interviewee no 13

The digital communication system also procured efficiency such as settling simple cases easily, relieving the telephone service of those minor issues that can be resolved by a single message turnaround. The opportunity for patients to raise several issues in 1 chat was considered good service, as was enabling patients to contact primary care at a time and place suiting them, with sufficient time to phrase their issues in a private setting.

You can see from the extent of the text, the way they write. It gives you a sense that this isn’t simply a matter of that prescription really.

Interviewee no 9

The digital communication system facilitated an opportunity to read the anamnesis, to prepare the response or a further chat with the patient, and to sustain preliminary consultations before the response, thus *procuring a preliminary plan* and giving a more accurate reply to the patient. This required that the staff rendered a full anamnesis, where templates were further expanded by means of free text. The photos that patients (or if the patient was a minor, a parent) could upload onto the digital communication system provided supplementary data facilitating *safer assessments* that could be agreed upon by the health care team during the triage process. The same questions were asked of all patients with a similar issue or symptom, and (for those centers using this routine) the same staff followed up on the patient’s chat.

You’re likely to have quite an extensive history, which you can read, and you can read the previous records before making further contact [in the chat]. When you get them on the phone, you have no idea why they’re calling and no chance to prepare prior to the dialogue.

Interviewee no 15

To some extent, the digital communication system thus *preserved resources*: although the innovation was time consuming, its implementation could lead to altered routines that provided the nurses with time to maintain communication. Although further engaging a particular professional group, having the patient anamnesis in place could procure a more accurate assessment, along with facilitating a nurse-physician interaction during triage.

### Summary

For health care staff, providing good service is key. This implies balancing the workload that an additional contact route imposes on one’s chores with patients’ positive feelings; many patients today anticipate the opportunity to engage in digital communication, although for other patients it is not an option. Digital communication poses both benefits and drawbacks: while issues can be managed more easily and with greater accuracy because of digital transmission, it requires the patient to have a certain level of both digital literacy and health literacy, including a basic understanding of health care services.

Staff need to find the time and routines to work around the obstacles presented by the innovation, determining how best to manage both the communication and the transfer of information to the patient record system. A team effort is helpful, starting with a joint decision to trial and implement the service. On the other hand, a lack of engagement or a lack of resources hampers the potential benefits of the digital communication system, even though it aids a more convenient service for some patient issues. The implementation of a digital system is shaped by the balance, or lack thereof, between staff workload and patient needs, and the competence among both patients and staff.

## Discussion

### Principal Findings

Corresponding to previous research, our study illustrated a future form of primary care in which digital communication services function as valuable complements, even though certain elements need further attention for optimization.

A concern raised both in our study and by others is the potential misuse of the service as a way to ensure speedier contact or a visit to the general practitioner. The staff posed this as potentially jeopardizing the ethical principles of health care delivery, such as directing resources to those most in need or with the greatest benefits, rather than those with a strong voice [17]. A similar risk of inequity is the challenges faced by patients who are not able to express themselves in writing: the staff valued a summary of the patient’s current health status and reason for contact before further communication, yet the anamnesis was sometimes incomplete. To aid safe assessments requires further guidance; additional instructions for patients would support not only their provision of details but also health literacy, of benefit to digital communication in primary care and beyond [18].

The notion that the inadequacies of digital communication are better settled by telephone [2,19] was addressed. While the chat was mainly based on text, face-to-face or telephone consultations have been found to provide additional information, enabling an appropriate assessment for patients who may not be able to express their problems adequately in writing [2]. The findings suggested that the digital communication service was best suited to less complex matters, but other digital solutions, such as video calls, have been found to be suitable in, for example, mental health issues [20]. Thus, online consultations may not be a replacement for, but rather a supplement to, traditional care [8] and should be part of an overall digital transformation of primary care, with more opportunities for self-administration [11].

Obtaining a further understanding of how to change and optimize both patient and staff behaviors regarding new digital forms of communication is warranted. Certain competencies required by the staff extend beyond their medical expertise to sustain a safe and person-centered approach to digital communication systems [21]. Voluntary testing of a digital communication system can derive from a joint or a management decision; staff readiness will lead to context variance, as will the engagement of either a few or all professionals [22]. In this study, implementation was facilitated primarily by means of training, a strategy known to transfer knowledge while having only potentially fair but often mixed effects on changing ways of doing things [23]. While in the last few decades understanding of the dynamics of implementation has increased, it is still fairly common for it to be mixed up with dissemination—that is, as primarily being the directed communication of information to increase knowledge and skills [24]. Rather, the complexity of how to support the satisfactory uptake of an innovation, to the extent that it actually becomes daily routine, requires further attention.

The Consolidated Framework for Implementation Research in particular echoes the professional end users’ employment of the digital communication system in primary care, suggesting that any innovation will be adapted as a result of the implementation process, the context in which it is used, and the users involved [22]. Our findings recognize a further need to evaluate eHealth implementation in primary care, incorporating constructs of importance [25], and emphasizing the significance of continuing to facilitate the implementation after the initial training when the eHealth intervention is launched. However, with little or no support for the implementation per se, the staff had settled upon routines themselves, although they lacked the resources needed to benefit from the innovation as teams or individual health professionals. Further enterprises engaging implementation champions would be likely to facilitate a more comprehensive process [25], including the mapping of needs and resources in context [26]. Altogether, further services may be needed, such as video consultations online, to expand the use of digital communication systems to a larger percentage of patients seeking primary care [27]. While staff members favor means that give patients a sense that the service is client centered and appreciate being up to date with other sectors in society [28], eHealth innovations that make sense in terms of quality and safety are likely to attract a more general uptake [19].

Recognizing the patient as a partner in matters pertaining to health and health care has become best practice, although it is still a work in progress with several models available [29]; shared decision making is one route to enhancing opportunities for patient participation, much like the standards for person-centered or patient-centered care. All in all, these create a mutual recognition of knowledge and experience between patients and health care professionals, including the recognition of patient preferences and health literacy—the latter in order to sustain a better understanding and engagement in health issues [30,31]. Digital tools, offering benefits such as the opportunity to describe one’s concerns, symptoms, and health issues at a time, place, and pace that suit oneself, appear on current evidence to be good for enhanced values in primary care [32]. Study findings indicate that staff favored patients’ appreciation and were willing to walk that extra mile to meet patient expectations, much like health care professionals who cautiously embrace digital have been found to favor increased efficiency, besides the prospect of providing patients with alternatives that align with their preferences [11]. To the best of our knowledge, this aspect has not been widely recognized as an incentive for facilitating implementation, but we suggest that ethics should be further investigated as a means for understanding such processes.

Our findings signify that, to staff, the digital communication service serves certain types of contacts better than others, but patients need further guidance as to which route is most appropriate for which issue. A potential means to enhance digital services and optimize their use by patients is to include patient representatives during both development and implementation projects [33]. Such enterprises, as well as everyday practice in the digital era, need to address the potential injustice between resourceful and frail individuals and groups in society [34]. The increased risk of inequity due to digital communication services renders a need for innovations that are easier to use and more effective than current alternatives [35].

### Limitations

While this study adds to the growing body of understanding of eHealth in primary care by proposing the further dimension of the importance of justifications for implementation, it was based on data collected in 5 primary care centers that had all volunteered to take part in a pilot test. Although the findings illustrate the complexity of implementation enterprises, the inclusion of more centers, across the entire spectrum of attitudes toward digital communication services, could have yielded additional input [36], as could potentially the inclusion of staff not engaged in the use of the digital service, particularly in terms of barriers to implementation of digital communication systems in primary care.

### Conclusion

A digital communication system may be simple enough to grasp but still present challenges requiring attention and in-house solutions in order to master it in daily practice. The health care professionals in this study considered the digital communication system to be more or less appropriate for different patients, but it may have aided the primary care nurse or team to settle certain issues more easily—or it may have created more problems than a telephone conversation. However, the possibility to see photographs and the opportunity to master the patient anamnesis prior to further contact made the primary care triage better and more efficient.

For an innovation such as a digital communication system to pay off in regular primary care, the implementation process should entail joint team engagement, with the readiness, resources, skills, and mandate to change routines as necessary. Patients need to use the service consciously for appropriate issues. By means of careful strategies, further systematic clinical and research efforts can better understand what works, where, for whom, how, and why—or why not. While staff members valued a digital communication system that is considered a good service by patients, ethical ideals should be considered when implementing digital communication systems in the primary care context.

## Appendix

Multimedia Appendix 1 Overview of global elements, categories, and subcategories.
