# Supplementary material for: Health Care Professionals’ Experience of a Digital Tool for Patient Exchange, Anamnesis, and Triage in Primary Care: Qualitative Study
Source: JMIR Hum Factors. 2020 Dec 14;7(4):e21698. doi: 10.2196/21698 (PMC7769692; doi:10.2196/21698)
Supplement: Multimedia Appendix 1 [file humanfactors_v7i4e21698_app1.doc]

Overview of the categories formed, their subsidiary subcategories, and the global themes serving the comprehensive understanding.

| **Global theme** | **Categories** | **Subcategories** |
| --- | --- | --- |
| the innovation | requiring particular skills from patients | entails working on an Internet-connected computer, iPad or smartphone |
|  |  | requires a basic perception of terms related to health and healthcare |
|  |  | affected by skills related to expressing oneself in writing |
|  | patients’ access to devices | requires access to an Internet-connected computer, iPad or smartphone |
|  |  | requires access to and skills to use e-ID |
|  | buttressing patient inequity | mainly beneficial for the young and/or resourceful |
|  |  | another route for contact |
|  |  | empowering those in society who can navigate the system |
|  | offering asynchronous chat | interpreted as a concurrent service |
|  |  | disparate limits for further dialogue |
| implementation – the device and the change process | varying pre-implementation process | training by the company, project instructor, or a colleague |
|  |  | in-house guidance or a joint event |
|  |  | the team being engaged in the dialogue re. the digital service pilot |
|  |  | a sole management election |
|  |  | management attitude engaging or ordering |
|  | manufactured for a team | simple to use |
|  |  | identifying routines |
|  |  | changing routines |
|  |  | setting up routines |
|  |  | triaging incoming cases |
|  |  | no corresponding increase in terms of staffing |
|  |  | nurses taking turns |
|  | a team effort | secretaries managing administrative issues |
|  |  | physicians managing complex medical issues |
|  |  | forwarding case to the most appropriate profession |
|  |  | physicians reluctant to engage |
|  | implementation takes time | beyond getting the system up and running |
|  |  | complex patient issues |
|  |  | ways to uphold safe procedures |
|  | inventing keys along the way | establishing structures scaffolding the context |
|  |  | changing routines |
|  |  | taking advantage of the innovation |
|  |  | pondering whether and how the innovation worked satisfactorily, or not |
| the barriers – patient and staff needs and skills | lacking synchronicity between systems | lacks connections |
|  |  | requires manual data registration |
|  |  | patients using several routes |
|  |  | multiple staff engaging with the same case |
|  | writing takes time | lacks templates for replies |
|  |  | writing takes longer than talking |
|  |  | time-lag |
|  | matters deriving from a wide range of aspects | difficult to chat with patients about mental health issues |
|  |  | permitting a more anonymous route to dialogue |
|  |  | verbal dialogue more beneficial |
|  | lacking overview | patients not having a set time-limit to respond |
|  |  | extending the communication |
|  |  | risky multitasking |
|  |  | assessing cases differently |
|  | missing data | patients not understanding the necessity for a complete anamnesis |
|  |  | skipping mandatory items |
|  |  | hearing a patient speak |
| the outcomes and pros – a work in progress | procuring safety | uploading of photos |
|  |  | safer and prompter triage with pictures |
|  |  | sustaining a safe environment |
|  | procuring efficiency | settling simple errands easily |
|  |  | concluding several issues in one chat |
|  | procuring a preliminary plan | reading the anamnesis, preparing the response |
|  |  | sustaining preliminary consultations |
|  | safer assessments | providing a full anamnesis, with templates further expanded by means of free text |
|  |  | supplementary data |
|  |  | agreed within the healthcare team |
|  |  | the same questions asked of all patients with a similar issue or symptom |
|  |  | the same staff follow-up on the patient’s chat |
|  | preserving resources | changing routines |
|  |  | procuring a more accurate assessment |
|  |  | facilitating nurse-physician interactions |
